# Bio-Alcohol Sensor Based on One-Dimensional Photonic Crystals for Detection of Organic Materials in Wastewater

**DOI:** 10.3390/ma15114012

**Published:** 2022-06-05

**Authors:** M. Al-Dossari, S. K. Awasthi, A. M. Mohamed, N. S. Abd El-Gawaad, W. Sabra, Arafa H. Aly

**Affiliations:** 1 Department of Physics, Faculty of Science, King Khalid University, Abha 62529, Saudi Arabia; 2 Department of Physics and Material Science and Engineering, Jaypee Institute of Information Technology, Noida 201304, India; 3 TH-PPM Group, Physics Department, Faculty of Sciences, Beni-Suef University, Beni Suef 62514, Egypt; 4 Faculty of Sciences, King Khalid University, Mohayel Asser, Abha 61421, Saudi Arabia

**Keywords:** photonic crystal, photonic band gap materials, organic material, biosensor

## Abstract

In this work, we have explored a novel application of one–dimensional (1D) photonic crystals (PCs) as a biomarker for the detection of organic materials in wastewater. The high concentration of organic materials may lead to adverse impact on human life. In order to save human life from these adverse effects, we have investigated the bio-alcohol sensing properties of a 1D multilayer periodic structure *(AB)^N^/C/(AB)^N^* capable of detecting organic materials in wastewater. The proposed structure works on the principle to detect a very small change in the refractive index of the wastewater sample under investigation by means of producing a shift in the position of the defect mode inside the photonic band gap (PBG) of the proposed structure. The transfer matrix method (TMM) has been used to investigate the transmission properties of the proposed design with the help of MATLAB software. We have also studied the effect of changes in the defect layer’s thickness, the volume fraction of the nanocomposite material and the incident angle on the sensitivity of our proposed bio-alcohol sensing design. Our bio-alcohol sensor shows a high sensitivity value of 500 nm/RIU and a low detection limit value of 1 × 10^−5^ RIU. The figure of merit and quality factor values of our bio-alcohol sensor are 5 × 10^3^ and 5.236 × 10^3^, respectively. The damping rate of the design is $\xi =95.4927\times 10^{-5}$.

## 1. Introduction

Nowadays, the demand for biosensors that can detect physiological change by means of investigating samples containing bio ingredients has been increased tremendously. Such kinds of user-friendly biosensors utilize the concentration of substances and other parameters of biological systems to produce rapid and accurate results [1,2,3]. There are a variety of applications, such as in medical diagnostics, drug discovery, detection of pollutants and the food industry [4,5], in which these biosensors have found their commendable utilization. In recent decades, keeping water crises in mind, researchers have given their technological efforts to treat contaminated water and make it reusable because water is the main source of life on our earth and we have limited sources of drinkable water. Actually, heavy water utilization is required in the industries associated with breweries and carbonated beverages, sugar mills, refineries associated with petroleum products, textile manufacturing, synthesis, organic chemicals, automobiles and aircraft and many other fields. These industries utilize freshwater to drain out waste along with several contaminants such as methanol, ethanol, propanol, acetone, butanol, chloroform, phenol, pesticides and herbicides [6,7] from their plants. This wastewater from various sources reaches to the marine environment through estuary, which is the area of confluence between land water and seawater. This mixing of land water with seawater results in global warming and also contaminates ground water reservoirs. Presently, the detection and analysis of anions and metal ions with the help of organic receptors is also one of the emerging areas of research in fields such as the environmental, chemical and biological sciences due to their involvement in human life. For example, Kurkuri et al. have suggested a sampling method for colorimetric detection of fluoride and iron ions in water by using their low-cost and user-friendly kit [8]. Additionally, Kurkuria et al. also suggested a chemodosimeter which could easily detect and remove traces of environmental hazard mercury ions from aqueous solutions [9]. Hence, it is our moral responsibility to develop effective technologies by which the contamination of pollutants into freshwater can be restricted. The excess presence of pollutants, especially due to organic waste into freshwater, may increase the level of their concentration, which may become harmful for human life due to their adverse effect. The degradation of organic material waste such as acetone, phenol, methanol, etc., from wastewater can be completed with the help of well-known conventional and unconventional methods [10,11]. The photonic and plasmonic biosensors may also be efficiently used for the degradation of organic waste from water waste to make it reusable.

PCs are the multilayer periodic structures which consist of two or more materials of different dielectric properties [12,13]. PCs have received more attention from researchers [14,15,16] due to their tremendous ability of controlling wave propagation by virtue of their unique property of having PBG [17]. The propagation of waves of specific frequencies which lie inside the PBG is forbidden due to Bragg scattering at each interface [18,19]. The PBG is also highly sensitive to the dielectric constant of their constituents. Biosensors composed of 1D PCs are used in many fields such as the detection of pollutants, biomedicine and biochemical engineering. Such biosensors have highly sensitivity [20,21] due to the effective and controlled interaction of electromagnetic waves (EMWs) with the analysts under investigation. The introduction of a defect layer into PCs results in a break in the periodicity of the structure, which ensures the presence of defect mode of high transmittance inside the PBG of the structure. This defect mode can be shifted either right or left inside the PBG by changing the structural parameters such as the refractive index of analyte, angle of incidence and thickness of the defect layer region. To improve the performance of photonic biosensors, scientists have utilized different types of constituent materials to design PCs such as superconductors, metal, left hand material (LHM) and nanoparticles. Unfortunately, metals are highly absorptive when the frequency of electromagnetic radiation falling on them is less than the plasma frequency of the metal. Superconductors are operating at specific conditions and temperatures whereas the fabrication of PCs composed of LHMs is limited only in the GHz range of the electromagnetic spectrum [22]. Recently, researchers have focused their attention on using nanocomposite materials in which metal nanoparticles are embedded. Photonic biosensors consisting of metal-embedded nanocomposite materials have low absorption in comparison to the metal-based photonic biosensing designs, wherein the metal nanosphere is immersed in to dielectric material [23] to form nanocomposite materials. Such PCs show new PBGs in the region of the plasma frequency of metal and are very sensitive to the nature of the polarization of incident light [24]. Herein, we utilize the transfer matrix method (TMM) to investigate the optical properties of the proposed bio-alcohol sensor composed of 1D PC with a defect to detect the water samples containing various kinds of organic materials. In this research work, the tuning of defect mode inside the PBG of the proposed structure is loaded separately with water samples containing methanol, acetone, ethanol, propanol, butanol, pentanol, chloroform and phenol, each of a concentration of 30% with respect to the pure water sample that has been studied. In order to induce the tunability, we have changed the defect layer’s thickness, the incident angle, the volume fraction of the nanocomposite material and also the concentration of organic material in the water sample. This article has four sections. The introduction is presented in Section 1. The architecture and theoretical analysis of the problem are given in Section 2. The results and discussions pertaining to the work have been discussed in Section 3. Finally, conclusions are discussed in Section 4.

## 2. The Architecture and Theoretical Analysis of the Problem

**Figure 1:**
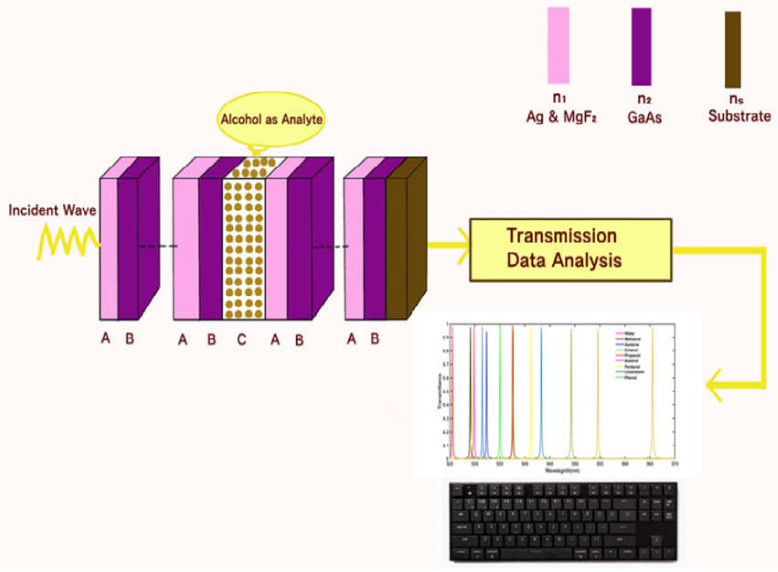
The architecture of the proposed 1D bio-alcohol sensor composed of 1D PCs with defect layer C sandwiched between the two layers B and A.

The proposed 1D PC with defect *(AB)^N^C(AB)^N^* is composed of two 1D conventional PC *(AB)^N^* which are associated with defect layer C of air, as shown in Figure 1 below. The cavity layer C is loaded one by one with different alcohol samples under investigation. The alphabets A and B of the design are used to represent nanocomposite material Ag&MgF_2_ and GaAs, respectively. The letter N is used to represent the period number of conventional 1D PC *(AB)^N^*. The thickness of layers A, B and C of the structure are represented as *d*_1_, *d*_2_ and *d_c_*, respectively. The refractive index of layers A, B and C are designated as *n*_1_, *n*_2_ and *n*_c_, respectively. We have assumed that the entire structure is immersed in the air of refractive index 1. The refractive index of the incident medium and substrate are represented by *n*_0_ and *n*_s_, respectively.

The refractive index of the nanocomposite material of permittivity *ε_eff_* is given by the Maxwell-Garnett formula as [25]:

(1) $$n_{1}=\sqrt{\varepsilon_{eff}}=\sqrt{\varepsilon_{d}\frac{\varepsilon_{m}+2\varepsilon_{d}+2f\left(\varepsilon_{m}-\varepsilon_{d}\right)}{\varepsilon_{m}+2\varepsilon_{d}-f\left(\varepsilon_{m}-\varepsilon_{d}\right)}}$$

where $\varepsilon_{d}$ is the permittivity of the dielectric host material, $\varepsilon_{m}$ is the permittivity of metal nanoparticles and f is the volume fraction of nanoparticles. According to the Drude model, the refractive index of silver is given by [26]

(2) $$n_{Ag}=\sqrt{\varepsilon_{Ag}}=\sqrt{\varepsilon_{0}-\frac{\omega_{P}^{2}}{\omega (\omega +i\gamma )}}$$

Here, *ε_0_*, *ω_P_* and *γ* are being used to represent the relative permittivity, plasma frequency and damping frequency of *Ag*, respectively. The damping frequency of *Ag* is further defined as [26]:

(3) $$\gamma =\gamma_{0}+q\frac{v_{f}}{a}$$

where *γ*_0_ is the decay constant, $v_{f}$ is the velocity of electrons at Fermi energy, *a* is the spherical radius of the metal nanoparticles and q is the electron scattering of nanoparticles, which has been taken as unity.

Next, we describe the detailed formulation of TMM to study the interaction between incident light and our design as presented in [27,28]:

(4) $$S=\left(\begin{array}{cc} S_{11} & S_{12}\\ S_{21} & S_{22} \end{array}\right)={\left(s_{1}s_{2}\right)}^{N}\left(s_{c}\right){\left(s_{1}s_{2}\right)}^{N}$$

Such that $S_{11}$, $S_{12}$, $S_{21}$ and $S_{22}$ are the elements of the total transfer matrix (*S*), N is the period number. $s_{1}$, $s_{2}$ and $s_{c}$ also represent the characteristic transfer matrix of layers A, B and C, respectively. These matrices are defined as

(5) $$s_{1}=\left(\begin{array}{cc} \cos\beta_{1} & \frac{-i\sin\beta_{1}}{p_{1}}\\ -ip_{1}\sin\beta_{1} & \cos\beta_{1} \end{array}\right)$$

(6) $$s_{2}=\left(\begin{array}{cc} \cos\beta_{2} & \frac{-i\sin\beta_{2}}{p_{2}}\\ -ip_{2}\sin\beta_{2} & \cos\beta_{2} \end{array}\right)$$

(7) $$s_{c}=\left(\begin{array}{cc} \cos\beta_{c} & \frac{-i\sin\beta_{c}}{p_{c}}\\ -ip_{c}\sin\beta_{c} & \cos\beta_{c} \end{array}\right)$$

Here $\beta_{1}=\frac{2\pi n_{1}d_{1}\cos\theta_{1}}{\lambda }$, $\beta_{2}=\frac{2\pi n_{2}d_{2}\cos\theta_{2}}{\lambda }$ and $\beta_{c}=\frac{2\pi n_{c}d_{c}\cos\theta_{c}}{\lambda }$ are used to represent phase difference in layers A, B and C respectively. 

For a transverse electric field (TE) waves, the value of $p_{i}=n_{i}\cos\theta_{i}$ (*i* = 1, 2 and *c* to represent layers A, B and C respectively). The ray angles $\theta_{1}$, $\theta_{2}$ and $\theta_{c}$ inside layers A, B and C respectively are related to the incident angle by using Snell’s law:

(8) $$n_{o}\sin\theta_{o}=n_{1}\sin\theta_{1}=n_{2}\sin\theta_{2}=n_{c}\sin\theta_{c}$$

For a 1D conventional PC of period *N*, we can use the second kind of Chebyshev polynomial to obtain the matrix ${\left(S_{1}S_{2}\right)}^{N}$. In light of the method elaborated above, we can easily find the total transfer matrix ${\left(S_{1}S_{2}\right)}^{N}Sc{\left(S_{1}S_{2}\right)}^{N}$ representing the entire structure. 

The transmission coefficient of 1D PC with defect *(AB)^N^C(AB)^N^* is given as

(9) $$t=\frac{2p_{o}}{\left(S_{11}+S_{12}p_{s}\right)p_{o}+\left(S_{21}+S_{22}p_{s}\right)}$$

where for TE wave $p_{o}=n_{o}\sin\theta_{o}$ for the incident medium and $p_{s}=n_{s}\sin\theta_{s}$ for the substrate.

The transmittance of the entire structure can be obtained by

(10) $$T=\frac{p_{s}}{p_{0}}{\left|t\right|}^{2}$$

## 3. Results and Discussion

This section of the manuscript deals with the results and discussion of the proposed work. The basic idea of our design is based on the existence of a resonant mode inside the PBG due to the break in the periodicity of the structure. The defect layer region is filled by various alcohol solutions of different concentrations under investigation separately, which results in the change in the refractive index of various alcohol samples as per the details mentioned in Table 1. This change in the refractive index of various alcohol samples causes the change in the position and intensity of the defect mode inside the PBG. In our proposed design, the numeric values of metal nanoparticles are taken as $\omega_{p}=1.365\times 10^{16}$ Hz, $\varepsilon_{0}=5$ [25,26], $\gamma_{0}=3.035\times 10^{13}$ Hz, *a* = 20 nm, $f=2\times 10^{-5}$ which are impeded in MgF_2_ of $\varepsilon_{d}=1.92$ [29]. The thickness of layers A, B and C are taken as *d*_1_ = 68.3 nm, *d*_2_ = 45.9 nm and *d_c_* = 125 nm, respectively. The period number has been fixed to 5. The refractive index of the substrate is taken as $n_{s}=1.5$. The proposed bio-alcohol sensor is composed of ten alternating layers of material nanocomposites and GaAs with an air cavity at the center.

The proposed structures can be realized by using a dip coating fabrication technique in addition to a sol-gel method because the spin coating process can allow the fabrication of a periodic layered structure composed of many materials including nanoparticles and polymer solutions. To initiate the spin coating process, a precursor solution is to be dropped over the flat substrate, then the solvent is evaporated from the substrate by conducting the spin process. Before spinning the next layer, we use an annealing procedure to solidify the earlier deposited layer on the substrate. The aforementioned process will be repeated till we obtain a stack of the required number of layers. The thickness of each film of the multilayer periodic stack can be controlled with the variation of either rotation speed or cast solution concentration. Since the sol-gel technique does not require any complex installations associated with technology, this minimizes the fabrication cost of the photonic structure.

After fabrication, the proposed structure is examined by using an angle-dependent spectroscopic elliposmetric method. This method helps to analyze the thickness and material dispersion in nanocomposite and GAaS material layers by using the software Complete EASE. The optical response of the proposed bio-alcohol sensor loaded with the water sample containing different organic materials is recorded by using the Kretschmann configuration. In this process, we have to connect the proposed structure and USB4000 spectrometer through the optical fiber via a microscope objective.

**Figure 2:**
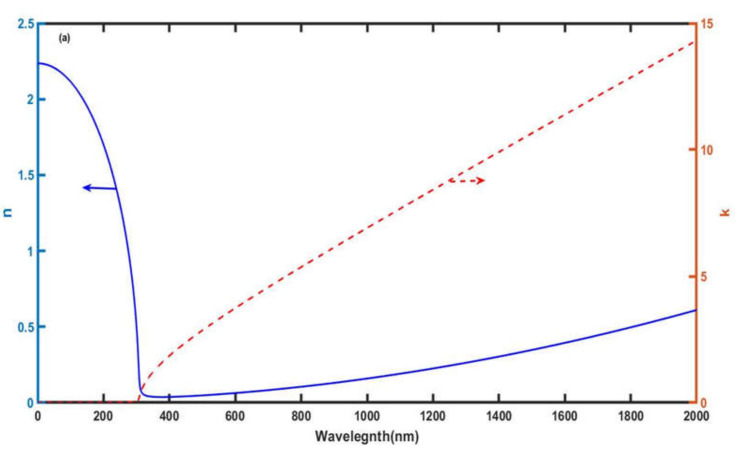

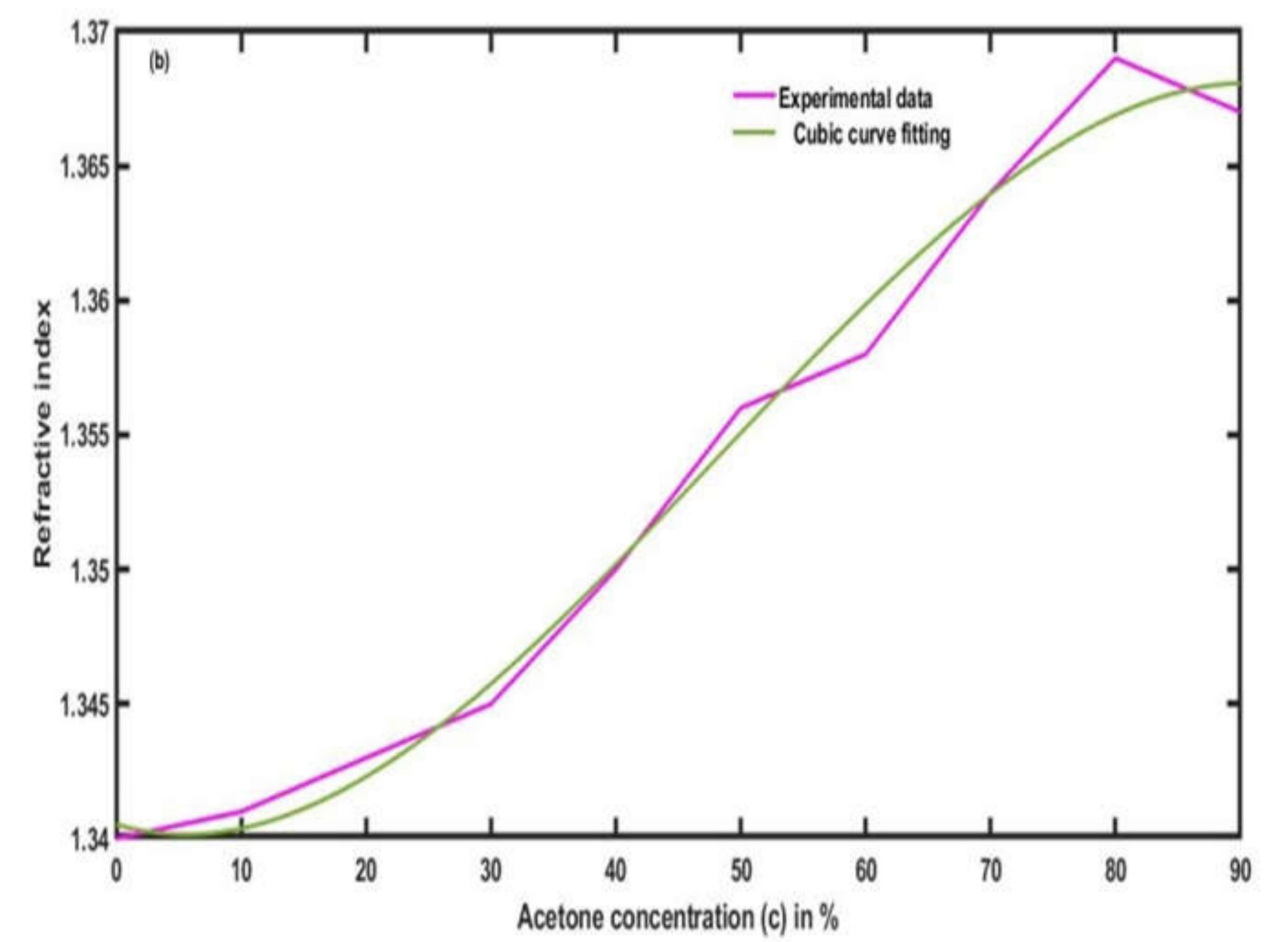
(**a**) The wavelength-dependent behavior of real (*n*) and imaginary (*k*) parts of the refractive index of silver (*n*_Ag_) and (**b**) The experimental and cubic curve fitting response showing the refractive index of water mixture (*n*_c_) dependent on acetone concentration.

Now, the wavelength-dependent nature of the real and imaginary part of the refractive index of silver has been discussed with the help of Figure 2a. This figure has been plotted with the help of Equation (2) in accordance with the Drude model. It shows the wavelength-dependent dispersive properties of the real and imaginary part of the refractive index of silver. It shows that in the visible region of the electromagnetic spectrum, the real part of the refractive index of silver is very small and varies little with respect to wavelength. On the other hand, the imaginary part of the refractive index of silver gradually increases and becomes significant with respective increases in the wavelength, as evident in Figure 2a. It shows that as wavelength increases from visible to infrared, the imaginary part of the refractive index of silver also increases from 0 to 14. It indicates the enhancement in losses associated with silver if we go from the visible to the infrared region. Next, we examine the change in the refractive index (*n_c_*) of water samples containing different acetone concentrations (*c*) as per the experimentally obtained data of Table 1, with the help of Abbe’s refractometer at a temperature of 30° and a wavelength of 589 nm. For this purpose, we have taken experimental data from reference [30] and applied cubic curve fitting to obtain the following relation (11), which elaborates the refractive index of water mixtures dependent on acetone concentration. The results are plotted in Figure 2b. Figure 2b shows the refractive index of the water mixture increases linearly with increase in the acetone concentration and nicely fitted over the experimental data of Table 1.

(11) $$n_{c}=1.3405-0.000137\;c+1.3106x10^{-5}c^{2}-9.0909x10^{-8}c^{3}$$

**Figure 3:**
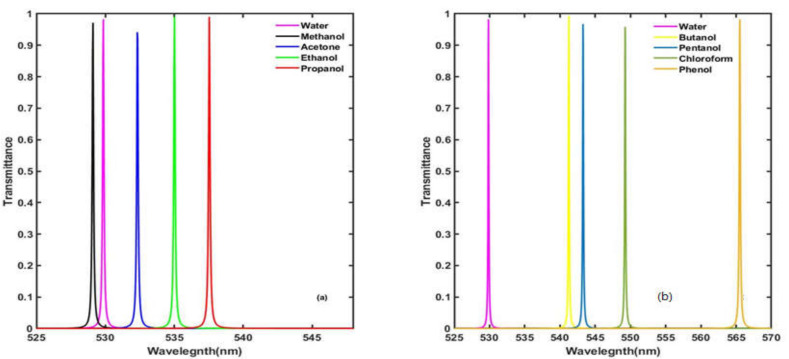
The transmittance spectra of the proposed bio-alcohol sensor with a cavity thickness *d_c_* = 125 nm at normal incidence when the cavity is loaded separately with water samples containing the organic materials (**a**) methanol, acetone, ethanol and propanol and (**b**) butanol, pentanol, chloroform and phenol of a concentration of 30% with respect to the pure water sample.

Now, we investigate the optical transmittance of the proposed design by loading the defect layer region one by one with different water samples containing the organic materials methanol, acetone, ethanol, propanol, butanol, pentanol, chloroform and phenol, each of a concentration *c* = 30%, and observe the corresponding change in the central wavelength and intensity of the respective defect modes inside the PBG of the proposed design with the help of Figure 3. It has been observed that, due to changes in the water samples containing methanol, acetone, ethanol, propanol, butanol, pentanol, chloroform and phenol, the central wavelength of the respective defect modes shifts to 529.1 nm, 532.3 nm, 535 nm, 537.6 nm, 541.3 nm, 543.3 nm, 549.3 nm and 565.5 nm inside the PBG with respect to the central wavelength of the defect mode corresponding to the pure water sample at 529.9 nm. The change in the central wavelength of the defect mode is due to change in the refractive index of the water samples containing pure water, methanol, acetone, ethanol, propanol, butanol, pentanol, chloroform and phenol in accordance with Table 2. The refractive index values of water samples containing methanol, acetone, ethanol, propanol, butanol, pentanol, chloroform and phenol, each of a concentration *c* = 30%, are 1.33, 1.3256, 1.3602, 1.3750, 1.3968, 1.4087, 1.444 and 1.542 respectively. The intensity of each defect mode is about 99%, as is evident from Figure 3a,b. The shifting of these defect modes corresponding to water samples containing different organic materials towards the higher-wavelength side inside the PBG is in accordance with the standing wave formulation of laser cavities [31]:

(12) $$\delta =l\lambda =n_{eff}\Delta d$$

where δ is the optical path difference, l is an integer, λ is the wavelength of incident light, $n_{eff}$ is the effective refractive index of the proposed bio-alcohol sensor and Δd is the geometrical path difference. The increase in the refractive index of water samples containing different organic materials results in the shifting of the defect mode towards the longer-wavelength side to keep the optical path difference fixed.

### 3.1. Effect of Increasing the Thickness of Defect Layer Region at θ = 0°

**Figure 4:**
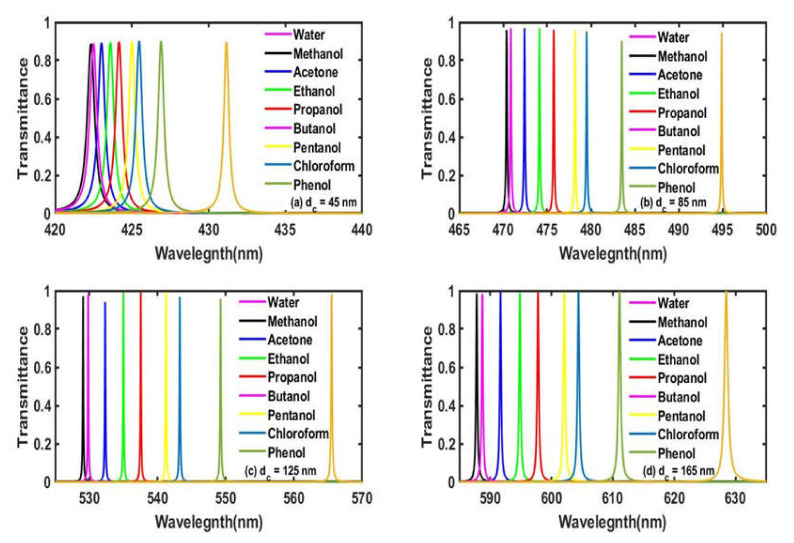
The transmittance spectra of the proposed bio-alcohol sensor loaded separately with water samples containing methanol, acetone, ethanol, propanol, butanol, pentanol, chloroform and phenol, each of a concentration of 30% with respect to pure water samples at a normal incidence corresponding to different cavity thicknesses (**a**) *d_c_* = 65 nm, (**b**) *d_c_* = 85 nm, (**c**) *d_c_* = 125 nm and (**d**) *d_c_* = 165 nm.

Now we have given our efforts to study the effect of increasing the thickness of the defect layer region from 45 nm to 165 nm in steps of 40 nm on the resonant peak inside the PBG corresponding to water samples containing different organic materials of a fixed concentration of 30%, which varies from methanol to phenol in accordance with Table 2. The transmission spectra of the proposed structure with four different defect layer thicknesses *d_c_* = 65 nm, 85 nm, 125 nm and 165 nm are plotted in Figure 4a–d, respectively with the angle, *θ* = 0°. The increase in the thickness of the defect layer region also increases the path by which it has to travel by electromagnetic waves inside the defect layer, which results in the strong interaction between the water sample under investigation and light. We have noticed from Figure 4a–d that the increase in the thickness of the defect layer also improvers the intensity as well as the full width half maximum (FWHM) of the defect mode corresponding to each water sample. At *d_c_* = 65 nm, the defect modes corresponding to the water sample containing methanol and phenol are located between the range 422.3 nm to 431.3 nm. The intensity of the defect modes is around 88%, and their FWHM is quite large. As we increase the thickness of the cavity region from 65 nm to 165 nm, we observe that their intensity starts to improve and reaches unity at *d_c_* = 165 nm. The increase in the thickness also reduces the FWHM of each defect mode, which is desirable for high-performance biosensors. At *d_c_* = 165 nm, the defect modes relocate their positions between 587.9 nm and 628.6 nm inside the PBG corresponding to the water samples containing methanol and phenol, respectively, as shown in Figure 4d. Thus, the defect layer’s thickness is one of the important parameters in designing any efficient biosensor, which also improves the sharpness of the tunneling peak inside the PBG.

### 3.2. Effect of Increasing the Incident Angle with d_c_ = 125 nm

**Figure 5:**
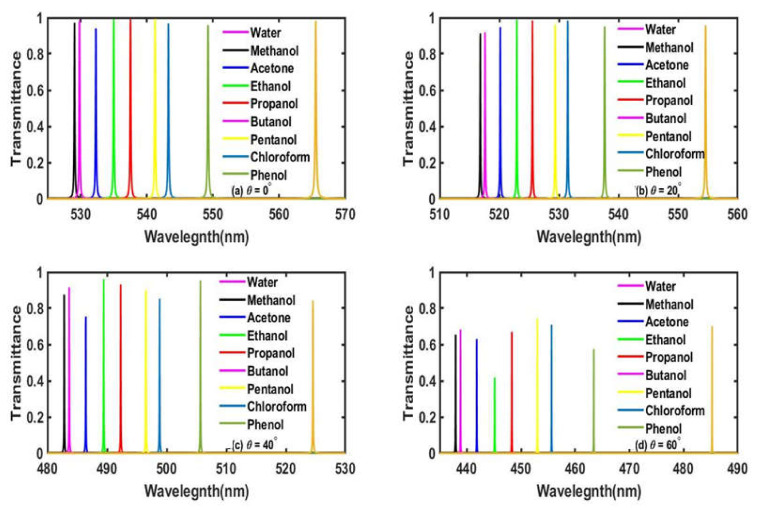
The transmittance spectra of the proposed bio-alcohol sensor loaded separately with water samples containing methanol, acetone, ethanol, propanol, butanol, pentanol, chloroform and phenol, each of a concentration of 30% with respect to the pure water sample at *d_c_* = 125 nm, corresponding to different incident angles (**a**) *θ* = 0° nm, (**b**) *θ* = 20°, (**c**) *θ* = 40° and (**d**) *θ* = 60°.

Next, we study the effect of changes in the angle of incidence corresponding to the TE polarization case on the transmission properties of the proposed structure at a fixed cavity thickness *d_c_* = 125 nm. All the other structural parameters have remained fixed as discussed above. The transmittance of the proposed bio-alcohol sensor loaded with water samples containing different organic materials included in this study corresponding to incidence angles 0°, 20°, 40° and 60° have been plotted in Figure 5a–d, respectively. Figure 5 shows that as the angle of incidence increases from 0° to 60° in steps of 20°, the defect modes associated with different organic samples start to move towards the lower-wavelength side with respect to the defect mode corresponding to the pure water sample due to the blue shifting of the PBG. The angle-dependent movement of these defect modes associated with water samples containing different organic materials along with the PBG can be easily explained on the basis of Bragg–Snell’s law [32,33] as

(13) $$m\lambda =2d\sqrt{n_{eff}^{2}-\sin^{2}\theta }$$

where λ is the wavelength of the incident light, m is the order of diffraction, d is representing interplanar distance, $n_{eff}$ is the effective refractive index and θ is the angle of the incident light. It can be easily understood from Equation (13) that the increase in the incident angle is compensated by the lowering of the central wavelength of the defect mode in order to maintain equality. Besides the movement of the defect mode dependent upon the incident angle, we have also noticed the gradual decrease in the intensity of defect modes with increases in the incident angles. At extremely higher angles, the decrease in the intensity of the defect modes is more prominent. Additionally, we have also observed the gradual reduction in the FWHM of each defect mode with an increase in the angle of incidence, which is always desirable for any high=performance biosensor.

### 3.3. Effect of Increasing the Volume Fraction of Ag Nanoparticles of Nano Composite Material Layers with d_c_ = 125 nm and θ = 0°

**Figure 6:**
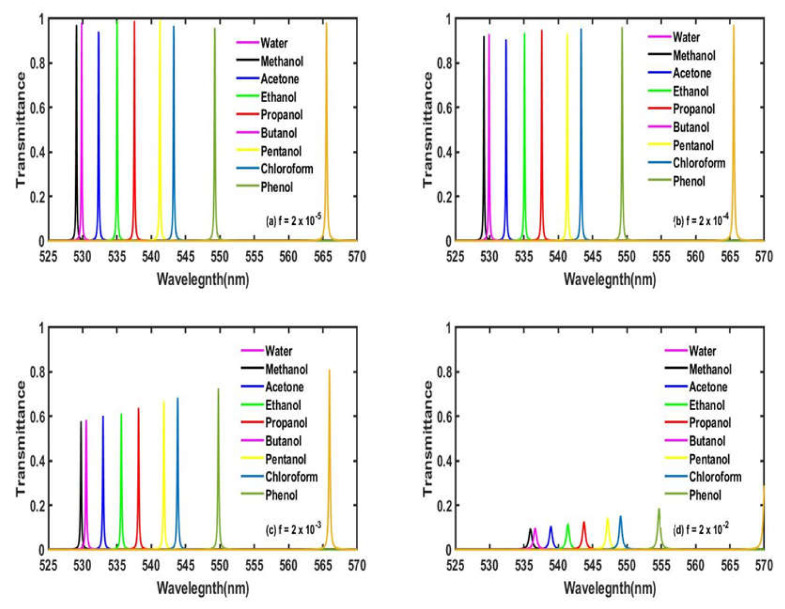
The transmittance spectra of the proposed bio-alcohol sensor loaded separately with water samples containing methanol, acetone, ethanol, propanol, butanol, pentanol, chloroform and phenol, each of a concentration of 30% with respect to pure water samples at a normal incidence with *d_c_* = 125 nm corresponding to different values of the volume fraction of Ag nanoparticles of nanocomposite material layers (**a**) *f* = 2 × 10^−5^, (**b**) *f =* 2 × 10^−4^, (**c**) *f =* 2 × 10^−3^ and (**d**) *f* = 2 × 10^−2^.

In this part, we have studied the effect of changes in the volume fraction (*f*) of silver nanoparticles embedded in the nanocomposite material on the transmittance spectra of our proposed bio-alcohol sensor design. For this purpose, we have selected four random values of *f* as 2 × 10^−5^, 2 × 10^−4^, 2 × 10^−3^ and 2 × 10^−2^. The transmission spectra corresponding to *f* = 2 × 10^−5^, 2 × 10^−4^, 2 × 10^−3^ and 2 × 10^−2^ have been plotted in Figure 6a–d, respectively. It has been found in Figure 6 that the increase in the volume fraction of silver leads to the decrease in the intensity of the defect modes corresponding to each water sample containing different organic materials. This is due to the fact that the presence of large numbers of Ag nanoparticles in the nanocomposite material layers enhances the absorption of light by the structure. This phenomenon becomes more prominent when the central frequency of the defect peak is closer to the plasma frequency of silver. Keeping this fact in mind, we have chosen $f=2\times 10^{-5}$, corresponding to which our designed bio-alcohol sensor shows least absorption.

### 3.4. Defining the Parameters for Evaluation of the Performance of the Proposed Bio-Alcohol Sensor 

In order to evaluate the performance of the proposed bio-alcohol sensor, we have calculated the most common parameters, which in turn examine the working performance of any biosensing structure. First, we have calculated the numeric value of sensitivity (*S*) for our design with the help of the following equation [34].

(14) $$S=\frac{\Delta \lambda }{\Delta n}$$

where, $\Delta \lambda =\lambda_{alcohols}-\lambda_{o}$, $\Delta n=n_{alcohols}-n_{o}$, $\lambda_{o}$ and $n_{o}$ are the wavelength and refractive index of pure water.

Next, we have calculated the quality factor (*Q*) value of our proposed design. One of the most essential requirements is to obtain accuracy in the measurements of biosensors. The higher value of the *Q* factor is always expected for any biosensing design to obtain accurate findings. The *Q* factor is defined [35] as

(15) $$Q=\frac{\lambda_{d}}{\lambda_{FWHM}}$$

where $\lambda_{d}$ is the central wavelength of the defect mode and $\lambda_{FWHM}$ is the *FWHM* of the defect mode.

The ability of any biosensor to sense the minute change in the position of the defect mode inside the PBG is associated with the figure of merit (*FoM*). It is inversely proportion to the FWHM of the defect mode and directly proportion to S according to the relation as under [36]

(16) $$FoM=\frac{S}{\lambda_{FWHM}}$$

The detection limit (*DL*), which describes the smallest detectable change in the index of refraction of the sample under examination, can be calculated by [37]

(17) $$DL=\frac{\lambda }{20SQ}$$

Finally, we have calculated the damping ratio (ζ), which is a dimensionless parameter. It describes how an oscillation in a system decays after a disturbance and is given by [37]

(18) $$\zeta =\frac{1}{2Q}$$

**Figure 7:**
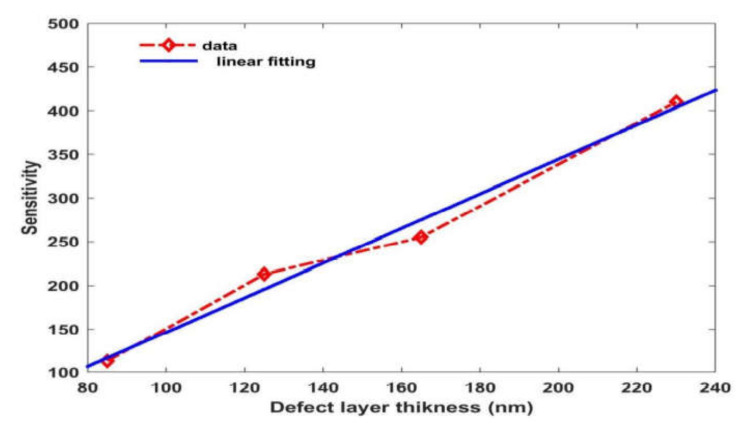
The sensitivity of the proposed bio-alcohol sensor as a function of the thickness of the defect layer when the cavity region is loaded with water samples containing phenol of a concentration of 30% at *θ* = 80°.

In order to analyze the dependence of the sensitivity of the proposed 1D bio-alcohol sensor as a function of the thickness of the defect layer region, we have plotted a sensitivity versus defect layer thickness diagram when the cavity region is loaded with the water sample containing phenol of a concentration of 30% at *θ* = 80°. Figure 7 shows that the variation between the changes in the sensitivity of the proposed bio-alcohol sensor from 113 nm/RIU to 405.66 nm/RIU is due to a change in the thickness of the defect layer region from 85 nm to 230 nm, respectively. We have also applied linear curve fitting on the simulated data to obtain the curve fitting equation $S(d_{c})=2.6634d_{c}-112.58$. The blue solid line curve in Figure 6 represents linear curve fitting between *S* and *d_c_*. Here, $R^{2}=0.998$ is the root mean square value between the simulated and linear fitting data. It indicates that the sensitivity increases linearly with increases in the thickness of the defect layer region.

### 3.5. Analysis of Bio-Alcohol Sensor for Achieving Optimum Performance 

The analysis of the proposed design has been carried out by means of calculating the numeric values of parameters *S*, *Q*, *FoM*, *DL* and ζ as defined in Section 3.4 above. Keeping in mind the importance of selecting the thickness of the cavity region as well as the angle of incidence as discussed in Section 3.1 and Section 3.2, respectively, to achieve our goal, we have tried two combinations to explore the optimum performance of the proposed design. First, we have fixed the angle of incidence at 70° and varied *d_c_* = 125 nm and 165 nm to obtain the numeric values of the parameters *S*, *Q*, *FoM*, *DL* and ζ. The results are summarized in Table 3 and Table 4, corresponding to *d_c_* = 125 nm and *d_c_* = 165 nm, respectively, at *θ* = 70°. Here, we have chosen *f* = 2 × 10^−5^ to optimize absorption, as discussed in Section 3.3. All the other parameters of the structure remain the same as discussed earlier.

Table 3 shows that the sensitivity of the design varies between 206.896 nm/RIU to 227.27 nm/RIU, corresponding to the water sample containing acetone and methanol, respectively, of a concentration of 30%. The order of the *Q*, *FoM* and ζ values of the proposed design with *d_c_* = 125 nm at *θ* = 70° are 10^2^ to 10^3^, 10^2^ and 10^−4^, respectively. Now, attempts have been made to improve the numeric values of these parameters. For this purpose, we have increased the dc to 165 nm, keeping *θ* = 70°. The numeric values of the parameters *S*, *Q*, *FoM* and ζ of the design with the modified value of *d_c_* = 165 nm are summarized in Table 4 below. 

We have seen from Table 4 that by increasing the thickness of the cavity region from 125 nm to 165 nm and keeping *θ* = 70°, one can easily improve the performance of the biosensor. Therefore, optimizing the thickness of the cavity region is a very essential requirement to design high-performance biosensing structures.

Next, we have further increased the thickness of the cavity region to 230 nm and varied the angle of incidence from 70° to 80° to study the performance of the proposed structure when it is loaded with water samples containing methanol, acetone, ethanol, propanol, butanol, pentanol, chloroform and phenol, each of a concentration of 30% with respect to the pure water sample. The numeric values of the parameters *S*, *Q*, *FoM* and ζ of the design with *d_c_* = 230 nm corresponding to *θ* = 70° to *θ* = 80° are summarized in Table 5 and Table 6, respectively.

It can be seen from Table 5 that as we increase the thickness from 165 nm to 230 nm and keep the angle of incidence fixed at 70°, the sensitivity of the proposed structure is tremendously enhanced. It varies between a maximum of 454.545 nm/RIU, corresponding to the water sample with methanol, to a minimum of 369.339 nm/RIU, corresponding to the water sample with phenol. Here, it should be noted that all calculations have been performed with respect to water. The other parameters of the design are also improved significantly. Finally, we have tried to improve the performance further by means of externally tuning the incident angle. For this reason, we have increased the angle of incidence to 80° and kept *d_c_* = 230 nm. The corresponding results are presented in Table 6 below.

**Figure 8:**
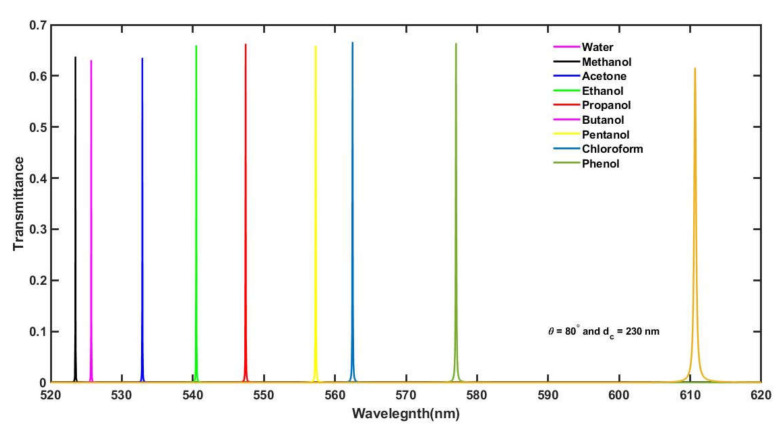
The transmittance spectra of the proposed bio-alcohol sensor loaded separately with water samples containing methanol, acetone, ethanol, propanol, butanol, pentanol, chloroform and phenol, each of a concentration of 30% with respect to the pure water sample at *θ* = 80° and *d_c_* = 230 nm.

From the data of Table 6, we can conclude that the sensitivity of the structure reaches its maximum under this combination and varies between a maximum of 500 nm/RIU and a minimum of 405.66 nm/RIU, corresponding to water samples containing methanol and phenol, each of a concentration of 30%, respectively. The order of the quality factor is 10^3^, which is large and signifies the accuracy of the measurements. The *FoM* of the design is also of the order 10^3^, which is as large as expected and indicates the ability of the design to sense the minute change in the position of the defect mode due to changes in the sample. The order of the damping ratio is very low, and hence the oscillations produced in the system due to changes in the water sample die out quickly. According to the current results, the average value of the detection limit is about 1 × 10^−5^ RIU, which is per our desire. The transmittance spectra of the proposed structure have been plotted in Figure 8 under optimum conditions as discussed above. It shows that, under optimum conditions, the defect modes corresponding to different water samples are found between 520 nm to 620 nm inside the PBG of the design. These defect modes are distinguishable and smooth. Though under optimum conditions the intensity of these defect modes reduces to 65%, this value is enough to be detected by transducers to produce measurable electric signals. There is one more common observation: as the refractive index of the water sample increases, the separation between consecutive defect modes also increases.

The comparison between the performance of the proposed bio-alcohol sensor and a previously reported similar kind of biosensors has been presented in Table 7. It shows that the sensitivity of our biosensor is better in contrast to the findings of previously reported biosensors. Moreover, the proposed bio-alcohol sensor has lots of additional advantages such as low cost, simpler design, high sensitivity, extremely low detection limit and easier fabrication and handling.

## 4. Conclusions

In the current work, we have proposed a novel bio-alcohol sensor capable of detecting different organic materials such as acetone, ethanol, methanol, propanol, butanol, pentanol, chloroform and phenol. The proposed 1D PC structure operates in the visible region of the electromagnetic spectrum. The transmission properties which account for the propagation of electromagnetic waves through the proposed 1D PC have been investigated by means of TMM and MATLAB software. How the change in the incident angle and thickness of the defect layer (alcohol) affects the performance of the proposed 1D bio-alcohol sensor has been estimated by theoretically calculating the sensitivity value of the design. It has been observed that the sensitivity of the proposed bio-alcohol sensor increases by increasing (1) the thickness of the defect layer region, (2) the incident angle and (3) the refractive index of the sample under investigation. The minute change in the refractive index of the alcohol samples under investigation from 1.3256 to 1.542 correspond to a change in the position of the central wavelength of the resonant defect mode inside the PBG from 523.6 to 611.9 nm. The maximum value of sensitivity and the minimum value of the detection limit of the proposed bio-alcohol sensor are found to be 500 nm/RIU and 1 × 10^−5^ RIU, respectively, which makes our bio-alcohol sensor suitable for high performance applications. Besides this, the figure of merit, quality factor and damping rate of our structure is 5.00 × 10^3^, 5.236 × 10^3^ and 95.4927 × 10^−5^, respectively. The high value for the figure of merit and the low value for the detection limit make our bio-alcohol sensor more sensitive, with a special ability to detect little changes in the refractive index of the sample under investigation.

## Figures and Tables

**Table 1:** The refractive index of different water mixtures of acetone concentration ranging from 0 to 90%.

Acetone Concentration (*c* in %)	Refractive Index of Water Mixture (*n_c_*)
0	1.340
10	1.341
20	1.343
30	1.345
40	1.350
50	1.356
60	1.358
70	1.364
80	1.367
90	1.368

**Table 2:** The refractive index of different water samples containing organic materials, each of a concentration of 30%.

Organic Materials	Refractive Index (*n_c_*)
Methanol	1.33
Acetone	1.3256
Ethanol	1.3602
Propanol	1.3750
Butanol	1.3968
Pentanol	.4087
Chloroform	1.444
Phenol	1.542

**Table 3:** Bio-alcohol sensor performance with *d_c_* = 125 nm corresponding to *θ* = 70°.

Sample Details	*n_c_*	*λ_c_* (nm)	*λ_FWHM_* (nm)	*S* (nm/RIU)	*Q*	*FoM*	$\zeta \times 10^{-4}$
Water	1.33	438.9	0.5	-	877.8	-	5.696
Methanol	1.3256	437.9	0.4	227.27	1094.75	568.175	4.5672
Acetone	1.3445	441.9	0.5	206.896	883.8	413.792	5.6573
Ethanol	1.3602	445.2	0.5	208.60	890.4	417.2	5.6154
Propanol	1.3750	448.4	0.5	211.11	896.8	422.22	5.5753
Butanol	1.3968	453	0.4	211.077	1132.5	527.6925	8.83002
Pentanol	1.4087	455.6	0.5	212.198	911.2	424.396	10.974
Chloroform	1.444	463.5	0.6	215.33	772.5	358.8833	6.4724
Phenol	1.542	485.4	0.4	219.33	1213.5	548.325	4.1203

**Table 4:** Bio-alcohol sensor performance with *d_c_* = 165 nm corresponding to *θ* = 70°.

Sample Details	*n_c_*	*λ_c_* (nm)	*λ_FWHM_* (nm)	*S* (nm/RIU)	*Q* × 103	*FoM* × 103	$\zeta \times 10^{-4}$
Water	1.33	489.1	0.1	-	4.891	-	1.022
Methanol	1.3256	487.7	0.2	318.18	2.4385	1.5909	2.0504
Acetone	1.3445	493.5	0.1	303.44	4.935	3.0344	1.01317
Ethanol	1.3602	498.4	0.2	308	2.492	1.53973	2.0064
Propanol	1.3750	502.9	0.2	306.666	2.5145	1.5333	1.988
Butanol	1.3968	509.6	0.1	307	5.096	3.0688	0.981169
Pentanol	1.4087	513.2	0.1	306.22	5.132	3.0622	0.974279
Chloroform	1.444	523.8	0.2	304.38	2.619	1.5219	1.90912
Phenol	1.542	551.5	0.2	294.33	2.7575	1.4716	1.813236

**Table 5:** Bio-alcohol sensor performance with *d_c_* = 230 nm corresponding to *θ* = 70°.

Sample Details	*n_c_*	*λ_c_* (nm)	*λ_FWHM_* (nm)	*S* (nm/RIU)	*Q* × 10^3^	*FoM* × 10^3^	$\zeta \times 10^{-4}$
Water	1.33	544.8	0.2	-	2.724	-	1.8355
Methanol	1.3256	542.8	0.2	454.545	2.714	2.27272	1.84229
Acetone	1.3445	551.3	0.2	448.27	2.7565	2.24135	2.23079
Ethanol	1.3602	558.3	0.2	447.019	2.7915	2.23595	2.23618
Propanol	1.3750	564.7	0.2	442.222	2.8235	2.21111	2.2613
Butanol	1.3968	573.5	0.3	429.644	1.91166	1.43214	3.4912
Pentanol	1.4087	578.2	0.3	424.396	1.92733	1.41465	3.5344
Chloroform	1.444	591.8	0.3	412.28	1.97266	1.37426	3.638
Phenol	1.542	623.1	0.8	369.339	0.77887	0.46167	10.8304

**Table 6:** Bio-alcohol sensor performance with *d_c_* = 230 nm corresponding to *θ* = 80°.

Sample Details	*n_c_*	*λ_c_* (nm)	*λ_FWHM_* (nm)	*S* (nm/RIU)	*Q* × 10^3^	*FoM* × 10^3^	$\zeta \times 10^{-4}$
Water	1.33	525.8	0.2	-	2.629	-	19.018
Methanol	1.3256	523.6	0.1	500	5.236	5.000	95.4927
Acetone	1.3445	533	0.1	496.55	5.330	4.9655	93.808
Ethanol	1.3602	540.6	0.1	490.06	5.406	4.9006	9.24898
Propanol	1.3750	547.7	0.1	486.66	5.477	4.8666	9.12908
Butanol	1.3968	557.5	0.1	474.55	5.575	4.7455	8.968
Pentanol	1.4087	562.9	0.3	471.41	1.8763	1.5713	26.6477
Chloroform	1.444	577.6	0.3	454.385	1.9253	1.51461	25.9695
Phenol	1.542	611.8	0.7	405.66	0.874	0.579514	57.208

**Table 7:** Comparison between the present sensor and previously reported sensors.

Year	Ref.	Structure	*S* (nm/RIU)	DL (RIU)	*FoM*
2008	[38]	Waveguide with microcavity	330	Not mention	Not mention
2011	[39]	PC waveguide	240	Not mention	Not mention
2011	[40]	PC slab waveguide	200	1 × 10^−3^	Not mention
2012	[41]	PC cavity biosensor	35	Not mention	Not mention
2013	[42]	PC waveguide	260	0.001	Not mention
2015	[43]	Waveguide with microcavity	425	0.001	Not mention
2019	[44]	2D PC based Bio-alcohol sensor	Not mention	Not mention	Not mention
2021	This work	1D PC based Bio-alcohol sensor	500	1 × 10^−5^	5 × 10^3^

## Data Availability

Not applicable.
